# Draft genome sequence of *Staphylococcus auricularis* PAPLE_T1 isolate of *Carica papaya* revealing biosynthetic clusters for beneficial metabolites

**DOI:** 10.1128/mra.01201-23

**Published:** 2024-03-08

**Authors:** Oyetayo Olaoluwa Adefiranye, Adetomiwa Ayodele Adeniji, Olajide Joseph Akinjogunla, Olayide Folashade Obidi, Ganiyu Oladunjoye Oyetibo, Matthew Olusoji Ilori, Olubukola Oluranti Babalola

**Affiliations:** 1 Department of Microbiology, Faculty of Science, University of Lagos, Lagos, Nigeria; 2 Centre for Epidemic Response and innovation (CERI), Tygerberg Campus, Stellenbosch University, Cape Town, South Africa; 3 Department of Microbiology, Faculty of Science, University of Uyo, Akwa Ibom, Nigeria; 4 Food Security and Safety Focus Area, Faculty of Natural and Agricultural Sciences, North-West University, Mmabatho, South Africa; University of Maryland School of Medicine, Baltimore, Maryland, USA

**Keywords:** agro-antibiotics, biotechnology, environmental reclamation, pathways and soil bioremediation

## Abstract

Genomic features of *Staphylococcus auricularis* PAPLE_T1 isolated from waste sample of *Carica papaya* obtained from Lagos State, Nigeria, revealed its putative capability to synthesize valuable secondary metabolites. *S. auricularis* PAPLE_T1 has a 2.4 Mb genome and could be useful as biological agro-antibiotics, for soil bioremediation and in biotechnological industry.

## ANNOUNCEMENT

Microbe-based production of valuable biotechnological products (1, 2) and environmental reclamation research have gained interest over the years (3–5). Members of the genus *Staphylococcus* have shown promise in the aforementioned research areas (6–8); however, few reports exist on biotechnological relevance of *Staphylococcus auricularis* (7, 9). *Staphylococcus auricularis* PAPLE_T1 was isolated from *Carica papaya* deteriorating samples obtained from Ikotun market, Lagos State, Nigeria (N 6° 27' 11.0016", E 3° 23' 44.9988"). *Carica papaya* waste flesh and peels (10 g) were rinsed, homogenized, and blended in sterile distilled water (100 mL) and then centrifuged at 250 r.p.m/2 mins. After serial dilution of the pellets, inoculated dilutions were 10^−4^, 10^−6^, and 10^−8^ in enrichment broth (yyeast extract 0.8%, glucose 1%, acetic acid 0.3%, ethanol 0.5%, peptone 1.5%) and distilled water (100 mL) for 3–7 days at pH 6.5. From each dilution, 0.1 mL was plated on Carr’s (yeast extract 3%, agar 2%, bromocresol green 0.002%, ethanol 2%) and Frateur’s media (yeast extract 10 g/L, ethanol 20 g/L, agar 20 g/L, calcium carbonate 20 g/L, and distilled water 1 L) and incubated for 3–7 days. Bacterial colonies were purified by streaking in fresh Frateur’s medium (10, 11). For useful insights on *S. auricularis* PAPLE_T1 intrinsic potentials, its entire genome was sequenced maintaining default parameters. Quick-DNA fungal/bacterial miniprep kit by Zymo Research, Irvine, CA, USA, was adopted in the extraction of genomic DNA from fresh glucose, yeast extract, and peptone broth at 28°C–30°C/48 h (12). Nextera XT Index Kit v2 (FC-131-2001–2004) was adopted for library preparation, while 2 × 150 bp paired-end sequencing was conducted using NexSeq 500 system (Illumina, San Diego, CA, USA). Trim Galore v0.6.6 was adopted for pre-assembly trimming (13). The quality of reads was ascertained with QUAST v5.0.2 (14). Assemblage of sequence reads into contigs was carried out with SPAdes v3.15.3 on KBase platform (15, 16). Contigs from KBase platform were uploaded on online servers of Bacterial and Viral Bioinformatics Resource Center (BV-BRC) (v3.30.19) (17), NCBI prokaryotic Genome Automatic Annotation Pipeline (PGAAP v4.2), and SEED Viewer v2.0 (18, 19) for automated annotation and comparison. Default settings were adopted for all bioinformatics analyses. Table 1 shows the genomic sequence data of *S. auricularis* PAPLE_T1. Pathosystems Resource Integration center (BV-BRC/PATRIC) online server revealed preliminary genome exploration of *S. auricularis* PAPLE_T1 to possess genes predicted to code for biosynthesis of agriculturally relevant and industrially valuable secondary metabolites like tetracycline [tetracycline biosynthesis pathway (ID 00253)], streptomycin [Streptomycin biosynthesis pathway (ID 00521)], carotenoid [carotenoid biosynthesis pathways (ID 00906)], and flavonoid [flavonoid biosynthesis pathway (ID 00941)]. The strain is predicted to have the potential of atrazine degradation by allophanate hydrolase 2 subunit 1 (EC 3.5.1.54; PID 00791), porphyrin and chlorophyll metabolism by adenosylcobinamide hydrolase (EC 3.5.1.90; PID 00860), as well as inositol phosphate metabolism by inositol-1-monophospsphatase (EC 3.1.3.25; PID 00562). Research integrating genome mining-guided microbe-based synthesis of important biotechnological products should benefit from the genome sequence of *S. auricularis* PAPLE_T1.

**TABLE 1:** Features of *Staphylococcus auricularis* PAPLE_T1 genome annotation^*a*^

Parameter	Value
Genome size	2,286,435 bp
Total number of reads	3,670,098 reads
Scaffolds number	16
Contigs number	19
tRNA	60
rRNA	10
Contig N50	208.9 Kb
Contig L50	3
GC percent	37
Genes	2,386
Protein coding	2,164
Plasmids	0
CDS^*b*^	2,269

^*a*^ NCBI Prokaryotic Genome Automatic Annotation Pipeline (PGAAP v6.5) (https://www.ncbi.nlm.nih.gov/datasets/genome/GCA_030418005.1/).

^*b*^ CDSs, coding sequences.

## Data Availability

The draft whole-genome shotgun project has been deposited at DDBJ/ENA/GenBank under the accession number JAUHQC000000000.1. The version described in this paper is version JAUHQC010000000, BioSample accession number SAMN36287968, BioProject accession number PRJNA990989, and Sequence Read Archive accession number SRR25949426.
